# Health Insurance

**Published:** 1917-03

**Authors:** 


**A Monthly Review of Medicine and Surgery**

EDITOR AND PUBLISHER, DR. A. L. BENEDICT, 228 Summer St., corner of Elmwood Ave., Buffalo, (Address for all communications. Please make personal and telephone calls before 1 P. M.)

Third, in age, on the western continent. Independent, but supports professional organizations. News limited mainly to Western N. Y. and Penn.

Subscription $2.00 a year; $3.00 for two years in advance. Changes and errors in address or failure or duplication of delivery, should be reported immediately.

Yearly Volume 72 MARCH, 1917 Number 8

### Health Insurance.

1. The proposed Compulsory Health Insurance Law, submitted with minor differences in over half the states, is obviously, a labor measure. To this we have no objection but:

2. Neither this nor any other legislation should give special privileges to a class, although the peculiar conditions applying to any occupation may fully justify legislation regarding it which would not be applicable to other occupations.

3. It is stated that the proposed law would, including dependents, apply to about 80% of the population. Whether this is true or not, and whether we view the matter from the interests of the community at large or of labor, there should be no arbitrary distinction between labor and work, in the broad sense of performing some necessary task in exchange for a living. We want no laboring class in this country, any more than we want a professional caste or a leisure class. The recognition of one implies the recognition of the other. Neither manual nor mental labor should be either favored or discriminated against. There is no form of muscular work that does not require brains while, per contra, there are remarkably few brain workers who do not, in the necessary course of their duties, use their muscles to a considerable degree. Whether muscular action involves high usage of calories and much sweat or whether it is of value mainly through its delicate coordination, is a matter of indifference. The present classification of labor, or at least what many advocate as a classification of labor, is almost purely arbitrary, either as to income, need for special protection or even the differentiation according to muscular or mental work. The law should provide equably for all honest workers, according to their need of special protection, without regard to whether their special danger is rheumatism from exposure or anaemia from indoor confinement, whether their working clothes are necessarily soiled or not, whether they are selling one kind of service or another, whether they are imagined to use their brains to direct their muscles or whether their intelligence must find some less direct method of interpretation into practical results.

4. From the practical standpoint, there is probably no better line of demarcation between those who need state insurance against disability and those who may safely be left to their own saving capacity or voluntary provision of insurance, than the one of income. But, it is a matter of common sense and justice that the clerk or salesman requires exactly the same degree of protection as the so-called manual laborer and that the high-salaried executive, nominally classified as a manual laborer, does not require special protection, any more than the man gaining the same income by teaching, preaching, practicing medicine or on an office force. The number of dependents upon a worker obviously should influence the income-line at which he is included within or excluded from the provisions of any such law and this fact incidentally brings up rather puzzling questions as to age and sex limits. It may be questioned whether the $1200 limit is a practicable one. It is not so long ago that such an income was regarded as indicating a pretty high degree of prosperity and, at least, sufficient ability to take care of one's own responsibilities. At least until the law has been tried out by experience, it is well to limit its application to an experimental basis. Serious difficulties have arisen in a western state by a miscalculation as to the funds necessary to provide the kind of insurance sought. A large deficit exists and the problem with regard to it is pressing and embarrassing in many ways. By setting an income limit that would cover a fair minority, say 25-30% of the population, that most needs protection, immediate requirements would be fulfilled, various practical problems could be solved without, on the one hand, the temptations to graft which an unnecessarily high premium estimate would tend to establish, or the serious deficit which would arise from an under-estimate of the expense. As suggested, the income limit should be in no sense arbitrary and should consider the dependents upon the worker.

5. We question seriously whether, beyond the requirement of safety and hygienic provisions, any part of the tax should fall directly on the employer. This view is based not on a consideration for the interests of the latter but on the inevitable tendency to place the burden ultimately on the insured, by lowering or at least failing to raise wages, and by raising prices which, in the long run, will have exactly the same tendency. Moreover, having unequivocally asserted that insurance should apply to all who need it, it is obvious that it will be exceedingly difficult in practice to distinguish between employers on a large scale and those on a small, even a domestic scale, and impossible to levy a proportionate tax on employers who are such merely by dealing with some one whose services are of value to them, but whom they employ only temporarily or indirectly. In short, the premiums must be borne by the beneficiaries except for such a percentage as may reasonably be contributed by the state as a philanthropic protection, not so much as a charity as for the ultimate best interests of the community.

6. As to the tendency to socialize the services of the medical profession, we regard the fears expressed as somewhat imaginary. With few exceptions, which the medical profession regrets as much as anyone else, its services always have been socialized in the practical sense. They have been free to the deserving poor, graded according to the means of the patient, never protected against even wilful neglect of compensation and never subject to general exploitation, according to business principles and procedures applying to services and commodities in general. As was pointed out in our issue of October 1915, 1.15% of the physicians of the country are employed by the nation; 2% of those of the state by the state; 0.5% of those of the county by the county; 3.7% of those of the city by the city. With due allowance for medical services regarding which statistics are very incomplete, 10% of the profession of the country is undoubtedly already in some form of public service and probably 20% of the aggregate income of the profession is publicly paid for, there being a great deal of partial public employment. Private organizations of various kinds, both corporate in the ordinary sense and philanthropic, also provide a considerable amount of the professional support, on a basis which is practically that implied in legislation of the kind considered. There are not—but there should be—comprehensive statistics on this point but we should be by no means surprised to learn that the profession of this country is already socialized to the extent of 40%, of its total earnings.

7. The real danger to the medical profession is not that it will be socialized by legislation of this sort, but that it will be exploited, as it undoubtedly has been in certain foreign countries, either by utterly inadequate pay or unreasonable requirements of work. It should be put squarely before labor leaders that the medical profession demands at least the pay of skilled labor, so far as practicable, the same insistence on hours of labor and the same allowance for night work or the undue fatigue of overtime. And it should be put squarely before legislators that the profession demands not merely the implied protection of representation on advisory boards but just as definite and authoritative protection of its interests as any other group of men involved.

